# Effects of nano particles on antigen-related airway inflammation in mice

**DOI:** 10.1186/1465-9921-6-106

**Published:** 2005-09-16

**Authors:** Ken-ichiro Inoue, Hirohisa Takano, Rie Yanagisawa, Miho Sakurai, Takamichi Ichinose, Kaori Sadakane, Toshikazu Yoshikawa

**Affiliations:** 1 Inhalation Toxicology and Pathophysiology Research Team, National Institute for Environmental Studies, Tsukuba, Ibaraki, Japan; 2 Inflammation and Immunology, Graduate School of Medical Science, Kyoto Prefectural University of Medicine, Kyoto, Japan; 3 Department of Health Science, Oita University of Nursing and Health Science, Oita, Japan

## Abstract

**Background:**

Particulate matter (PM) can exacerbate allergic airway diseases. Although health effects of PM with a diameter of less than 100 nm have been focused, few studies have elucidated the correlation between the sizes of particles and aggravation of allergic diseases. We investigated the effects of nano particles with a diameter of 14 nm or 56 nm on antigen-related airway inflammation.

**Methods:**

ICR mice were divided into six experimental groups. Vehicle, two sizes of carbon nano particles, ovalbumin (OVA), and OVA + nano particles were administered intratracheally. Cellular profile of bronchoalveolar lavage (BAL) fluid, lung histology, expression of cytokines, chemokines, and 8-hydroxy-2'-deoxyguanosine (8-OHdG), and immunoglobulin production were studied.

**Results:**

Nano particles with a diameter of 14 nm or 56 nm aggravated antigen-related airway inflammation characterized by infiltration of eosinophils, neutrophils, and mononuclear cells, and by an increase in the number of goblet cells in the bronchial epithelium. Nano particles with antigen increased protein levels of interleukin (IL)-5, IL-6, and IL-13, eotaxin, macrophage chemoattractant protein (MCP)-1, and regulated on activation and normal T cells expressed and secreted (RANTES) in the lung as compared with antigen alone. The formation of 8-OHdG, a proper marker of oxidative stress, was moderately induced by nano particles or antigen alone, and was markedly enhanced by antigen plus nano particles as compared with nano particles or antigen alone. The aggravation was more prominent with 14 nm of nano particles than with 56 nm of particles in overall trend. Particles with a diameter of 14 nm exhibited adjuvant activity for total IgE and antigen-specific IgG_1 _and IgE.

**Conclusion:**

Nano particles can aggravate antigen-related airway inflammation and immunoglobulin production, which is more prominent with smaller particles. The enhancement may be mediated, at least partly, by the increased local expression of IL-5 and eotaxin, and also by the modulated expression of IL-13, RANTES, MCP-1, and IL-6.

## Introduction

Previous epidemiological studies have indicated that long-term exposure to ambient particulate matter (PM) is linked to increases in mortality and morbidity related to respiratory diseases [1,2]. The concentration of PM of mass median aerodynamic diameter (a density-dependent unit of measure used to describe the diameter of the particle) < or 10 μm (PM10) is related to daily hospital admissions for asthma, acute and chronic bronchiolitis, and lower respiratory tract infections [3]. PM of mass median aerodynamic diameter < or 2.5 μm (PM2.5) are more closely associated with both acute and chronic respiratory effects and subsequent mortality than PM10 [4]. Our laboratory has researched health effects of diesel exhaust particles (DEP), main constituents of PM2.5 in urban areas, especially *in vivo*. We have reported that DEP exacerbate allergic asthma [5] and acute lung injury related to bacterial infection in murine models [6].

Recently, nano particles, particles less than 0.1 μm in mass median aerodynamic diameter, have been implicated to affect cardiopulmonary systems [4,7]. Indeed, two *in vivo *studies have demonstrated that nano particles induce prominent airway inflammation as compared with larger particles [8,9]. Nano particles which have a larger surface area than the particles with larger size are able to penetrate deeply into the respiratory tract and cause a greater inflammatory response [10,11].

Bronchial asthma has been recognized as chronic airway inflammation that is characterized by an increase in the number of activated lymphocytes and eosinophils. A number of studies have shown that various particles including carbon black (CB) can enhance allergic sensitization [12-14]. CB has been demonstrated to enhance proliferation of antibody forming cells and both IgE and IgG levels [15,16]. Ultrafine particles (PM and CB) reportedly exaggerate allergic airway inflammation *in vivo *[17,18]. However, all the studies have not described the size of particles they used. Therefore, no research has been addressed the size effects of particles or nano particles on allergic airway inflammation *in vivo*.

The aim of the present study was to elucidate the effects of two sizes of carbon nano particles (14 nm or 56 nm) on allergic airway inflammation, local expression of cytokines, chemokines, and 8-hydroxy-2'-deoxyguanosine (8-OHdG), and production of total IgE and antigen-specific IgG_1_, IgG_2a_, and IgE.

## Materials and methods

### Animals

Male ICR mice 6 to 7 wk of age and weighing 29 to 33 g (Japan Clea Co., Tokyo, Japan) were used in all experiments. They were fed a commercial diet (Japan Clea Co.) and given water ad libitum. Mice were housed in an animal facility that was maintained at 24 to 26°C with 55 to 75% humidity and a 12-h light/dark cycle.

### Study protocol

**Figure 1:**
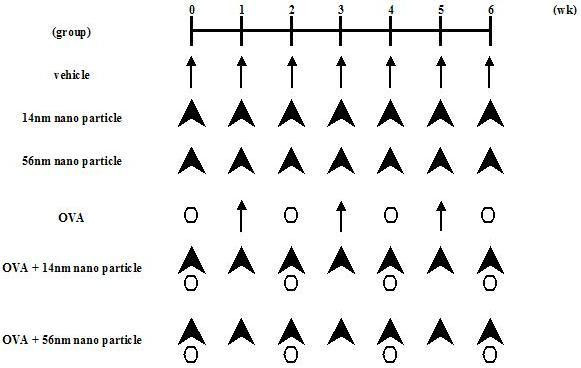
Study Protocol.

Mice were divided into six experimental groups (Fig. 1). The vehicle group received phosphate-buffered saline (PBS) at pH 7.4 (Nissui Pharmaceutical Co., Tokyo, Japan) containing 0.05% Tween 80 (Nakalai Tesque, Kyoto, Japan) once a week for 6 wk. The ovalbumin (OVA) group received 1 μg of OVA (Sigma Chemical, St. Louis, MO) dissolved in the same vehicle every 2 wk for 6 wk. The nano particle groups received 50 μg of nano particles (14 nm: PrinteX 90 or 56 nm: PrinteX 25, degussa, Dusseldorf, Germany) suspended in the same vehicle every week for 6 wk. The OVA + nano particle groups received the combined treatment in the same protocol as the OVA and the nano particle groups, respectively. The surface area of the 14 nm nano particles was 300 m^2^/g and that of 56 nm nano particles was 45 m^2^/g. The size of each particle was quantified by JEM-2010 transmission electron microscope (TEM; JEOL, Tokyo, Japan). Nano particles were autoclaved at 250°C for 2 h before use. The suspension was sonicated for 3 min using an Ultrasonic disrupter (UD-201; Tomy Seiko, Tokyo, Japan). In each group, vehicle, OVA, nano particles, or OVA + nano particles was dissolved in 0.1 ml aliquots, and inoculated by the intratracheal route through a polyethylene tube under anesthesia with 4% halothane (Hoechst, Japan, Tokyo, Japan). The animals were studied 24 h after the last intratracheal administration, with lung histology, bronchoalveolar lavage (BAL), protein levels of cytokines and chemokines in the lung tissue supernatants, immunohistochemistry for 8-OHdG, and with Igs. The studies adhered to the National Institutes of Health guidelines for the experimental use of animals. All animal studies were approved by the Institutional Review Board.

### Blood retrieval and analysis

Mice were anesthetized with diethyl ether. The chest and abdominal walls were opened, and blood was retrieved by cardiac puncture. Serum was prepared and frozen at – 80°C until assayed for total IgE and antigen-specific IgG_1_, IgG_2a_, and IgE.

### Histologic evaluation

After exsanguinations, the lungs were fixed by intratracheal instillation with 10% neutral phosphate-buffered formalin at a pressure of 20 cm H_2_O for at least 72 h. Slices 2 to 3 mm thick of all pulmonary lobes were embedded in paraffin. Sections 3 μm thick were stained with Diff-Quik (International Reagents Co., Kobe, Japan) or periodic acid-Schiff (PAS) and examined by two of us (HT and KI) in a blind fashion.

### Morphometric analysis for numbers of eosinophils, neutrophils, mononuclear cells, and goblet cells around the airways

Sections were stained with Diff-Quik to quantitate the numbers of infiltrated eosinophils, neutrophils, and mononuclear cells. The length of the basement membrane of the airways was measured by videomicrometer (Olympus, Tokyo, Japan) in each sample slide. The number of eosinophils, neutrophils, and mononuclear cells around the airways were counted with a micrometer under oil immersion. Results were expressed as the number of inflammatory cells per millimeter of basement membrane as described previously [5].

To quantitate goblet cells, sections were stained with PAS. The number of goblet cells in the bronchial epithelium was counted by micrometer. Results were expressed as the number of goblet cells per millimeter of basement membrane as described previously [5].

### BAL

The trachea was cannulated after the collection of blood. The lungs were lavaged with 1.2 ml of sterile saline at 37°C, instilled bilaterally by syringe. The lavage fluid was harvested by gentle aspiration. This procedure was conducted two more times. The average volume retrieved was 90 % of the 3.6 ml that was instilled; the amounts did not differ by treatment. The fluid collections were combined and cooled to 4°C. The lavage fluid was centrifuged at 300 g for 10 min, and the total cell count was determined on a fresh fluid specimen using a hemocytometer. Differential cell counts were assessed on cytologic preparations. Slides were prepared using an Autosmear (Sakura Seiki Co., Tokyo, Japan) and were stained with Diff-Quik (International reagents Co.). A total of 500 cells were counted under oil immersion microscopy.

### Quantitation of cytokine and chemokine protein levels in the lung tissue supernatants

In a separate series of experiments, the animals were exsanguinated and the lungs were subsequently homogenized with 10 mM potassium phosphate buffer (pH 7.4) containing 0.1 mM ethylenediaminetetraacetic acid (Sigma, St Louis MO), 0.1 mM phenylmethanesulphonyl fluoride (Nacalai Tesque, Kyoto, Japan), 1 μM pepstatin A (Peptide Institute, Osaka, Japan) and 2 μM leupeptin (Peptide Institute) as described previously [5]. The homogenates were then centrifuged at 105,000 g for 1 h. The supernatants were stored at -80°C. Enzyme-linked immunosobent assays (ELISA) for interleukin (IL)-4 (Amersham, Buckinghamshire, UK), IL-5 (Endogen, Cambridge, MA), IL-6 (Biosource, Nivelles, Belgium), IL-13, eotaxin, macrophage chemoattractant protein (MCP)-1, and regulated on activation and normal T cells expressed and secreted (RANTES: R&D systems, Minneapolis, MN) in the lung tissue supernatants were conducted using matching antibody pairs according to the manufacture's instruction. The second antibodies were conjugated to horseradish peroxidase. Subtractive reading of 550 nm from the reading at 450 nm were converted to pg/ml using values obtained from standard curves generated with varying concentrations of recombinant IL-4, IL-5, IL-6, IL-13, eotaxin, MCP-1, and RANTES, with limits of detection of 5 pg/ml, 5 pg/ml, 3 pg/ml, 1.5 pg/ml, 2 pg/ml 2 pg/ml, and 2 pg/ml, respectively.

### Immunohistochemistry

The production of 8-OHdG in the lung was detected by immunohistochemical analysis (n = 8 in each group) using anti-8-OHdG polyclonal antibody (Japan Institute for the Control of Aging, Shizuoka, Japan) as described previously [19,20]. Deparaffinized slides were blocked with 10% goat serum for 1 h. After blocking, anti-8-OHdG antibody (0.5 μg/ml) was incubated with the sections for 1 h at room temperature, followed by the incubation of a biotinylated secondary antibody and streptavidin-peroxidase conjugate. Then, the slides were incubated with 3-amino, 9-ethyl-carbazole chromogen, and counterstained with hematoxylin in AutoProbe III kit (Biomeda, Foster City, CA, USA). For each of the lung specimens, the extent and intensity of staining with anti-8-OHdG antibodies were graded on a scale of 0–4+ by two blinded observers on two separate occasions using coded slides as previously described [21]. A 4+ grade implies maximally intense staining, whereas 0 implies no staining.

### Antigen-specific IgG determination

Antigen-specific IgG_1 _or IgG_2a _antibodies were measured by ELISA with solid-phase antigen [5,22]. In brief, microplate wells (Dynatech, Chantilly, VA) were coated with OVA overnight at 4°C and then incubated at room temperature for 1 h with PBS containing 1% bovine serum albumin (BSA; Sigma) containing 0.01% thimerosal (Nakalai Tesque). After washing, diluted samples were introduced to the microplate and incubated at room temperature for 1 h. After another washing, the wells were incubated at room temperature for 1 h with biotinylated rabbit anti-mouse IgG_1 _or IgG_2a _(Zymed Laboratories, San Francisco, CA). After yet another washing, the wells were incubated with horseradish-peroxidase-conjugated streptavidin (Sigma) at room temperature for 1 h. The wells were then washed and incubated with o-phenylenediamine and H_2_O_2 _in dark at room temperature for 30 min. The enzyme reaction was stopped with 4 N H_2_SO_4_. Absorbance was read at 492 nm. Each plate incubated a previously screened standard plasma that contained a high titer of anti-OVA antibodies. The results were expressed in titers, calculated based on the titers of the standard plasma. Cut off values for antibody-positive plasma were set to hold as the mean value of absorbance of preimmune plasma.

### Total IgE and antigen-specific IgE determination

Antigen-specific IgE antibody was measured by IgE-capture ELISA [5,22]. In brief, microplate wells were coated with a rat anti-mouse IgE monoclonal antibody (Yamasa Syoyu Co., Chiba, Japan) at 37°C for 3 h and then incubated at 37°C for 1 h with 1% BSA-PBS and 0.01% thimerosal. After washing with PBS containing 0.05% Tween 20 (PBST; Nacalai Tesque), diluted samples were introduced to the microplate and incubated overnight at 4°C. After washing with PBST, biotinylated OVA was added to each well and incubated for 1 h at room temperature with β-D-galactosidase-conjugated streptavidin (Zymed). After the final washing, the wells were incubated with 4-methylumbelliferyl-β-galactoside (Sigma) as the enzyme substrate at 37°C for 2 h. The enzyme reaction was stopped with 0.1 M glycine-NaOH (pH, 10.3). The fluorescene intensity was read by a microplate fluorescene reader (Fluoroskan Flow Laboratories, Costa Mesa, CA). Each plate included a previously screened standard plasma that contained a high titer of anti-OVA antibodies. The results were expressed in titers, calculated based on the titers of the standard plasma. Cut off values for antibody-positive plasma were set two hold as mean fluorescene units of preimmune plasma. Total IgE was measured by capture ELISA in a manner similar to the detection of antigen-specific IgE. A biotinylated rat anti-mouse IgE (BD Biosciences Pharmingen, San Diego, CA) was used to detect captured IgE in place of biotinylated OVA. A_450 _readings of the samples were converted to nanograms per milliliter using a standard curve generated with double dilutions of mouse IgE κ isotype standard (BD Biosciences Pharmingen).

### Statistical analysis

Data were reported as mean ± SEM. Differences in the numbers of infiltrated inflammatory cells and goblet cells, cytokine protein levels, and immunogloblin concentrations and titers between groups were determined using analysis of variance (Stat view version 4.0; Abacus Concepts, Inc., Berkeley, CA) as described previously [5]. If differences between groups were significant (P < 0.05), Fisher's protected least significant difference test was used to distinguish between pairs of groups.

## Results

### Effects of nano particles on antigen-related airway inflammation

To evaluate the effect of nano particles on antigen-related airway inflammation, we investigated the cellular profile of BAL fluid and lung histology.

The numbers of total cells and macrophages were significantly greater in the nano particle, OVA, and OVA + nano particle groups than in the vehicle group (P < 0.01: Table 1). Furthermore, the numbers were significantly greater in the OVA + 14 nm nano particle group than in the OVA group or the 14 nm particle group (P < 0.01: Table 1). Although the numbers were greater in the OVA + 56 nm nano particle group than in the OVA group or the 56 nm nano particle group, the difference did not achieve significance. OVA challenge increased the number of eosinophils as compared with vehicle challenge without significance. The numbesr of eosinophils were greater in the OVA + nano particle groups than in the vehicle group (P < 0.05 for OVA + 14 nm nano particle, N. S. for OVA + 56 nm nano particle). The number was significantly greater in the OVA + 14 nm nano particle group than in the OVA group (P < 0.01) or 14 nm nano particle group (P < 0.05). The number was also greater in the OVA + 56 nm nano particle group than in the OVA group or 56 nm nano particle group, but the difference did not achieve significance. Challenge with nano particles significantly elevated the numbers of neutrophils as compared with vehicle challenge (P < 0.01 for 14 nm, P < 0.05 for 56 nm). OVA also elevated the number without significance as compared with vehicle challenge. The number was significantly greater in the OVA + 14 nm nano particle group than in the 14 nm nano particle group (P < 0.05) or the OVA group (P < 0.01). The number was also greater in the OVA + 56 nm nano particle group than in the nano particle group or the OVA group, the difference did not reach significance. Challenge with nano particles elevated the numbers of mononuclear cells as compared with vehicle challenge (P < 0.05 for 14 nm, N. S. for 56 nm). OVA also elevated the number of mononuclear cells without significance as compared with vehicle challenge. The number was significantly greater in the OVA + 14 nm nano particle group than in the vehicle (P < 0.01) or the OVA group (P < 0.05). The number was greater in the OVA + 56 nm nano particle group than in the OVA group, but difference did not achieve significance. There were no significant differences between the nano particle groups and OVA + nano particle groups.

**Table 1:** Cellular profile in bronchoalveolar lavage fluid.

Group	Animals *(n)*	Total Cells *(× 10^4^/total BAL)*	Macrophages *(× 10^4^/total BAL)*	Eosinophils *(× 10^4^/total BAL)*	Neutrophils *(× 10^4^/total BAL)*	Mononuclear Cells *(× 10^4^/total BAL)*
vehicle	16	36.88 ± 3.56	36.74 ± 3.53	0 ± 0	0.12 ± 0.05	0.015 ± 0.01
14 nm	13	111.69 ± 9.27**	83.79 ± 6.03**	0.332 ± 0.176	27.04 ± 4.98**	0.491 ± 0.201*
56 nm	14	97.36 ± 16.06**	88.64 ± 15.34**	0.331 ± 0.177	8.09 ± 2.49*	0.265 ± 0.093
OVA	16	85.06 ± 12.63**	81.91 ± 12.4**	0.705 ± 0.255	2.2 ± 0.62	0.121 ± 0.059
OVA + 14 nm	16	193.69 ± 18.33** ^## $$^	141.86 ± 14.97** ^## $^	13.667 ± 4.731** ^## $^	36.9 ± 3.67** ^## $^	0.878 ± 0.232** ^#^
OVA + 56 nm	17	102.65 ± 11.64**	90.7 ± 10.12**	3.984 ± 2.669	7.79 ± 2.29*	0.204 ± 0.073

Six groups of mice were intratracheally administered with vehicle, ovalbumin (OVA), nano particles, or combinations of OVA and nano particles for 6 wk. Bronchoalveolar lavage (BAL) was conducted 24 h after the last intratracheal instillation. Total cell counts were determined on fresh BAL fluid, and differential cell counts were assessed with Diff-Quik-staining. Results are presented as mean ± SEM. *P < 0.05 versus vehicle, **P < 0.01 versus vehicle, ^#^P < 0.05 versus OVA, ^##^P < 0.01 versus OVA. ^$^P < 0.05 versus nano particles. ^$$^P < 0.01 versus nano particles.

The magnitude and cellular profiles of airway inflammation were also evaluated in lung specimens stained with Diff-Quik. Intratracheal instillation of nano particles provided diffuse deposition of the particles into the bilateral lungs, including the bronchi and alveolar spaces. The particles were occasionally present within the subepithelial neutrophils and alveolar macrophages. The combined instillation of OVA + 14 nm nano particles for 6 wk led to a marked infiltration of eosinophils and mononuclear cells around the bronchi and bronchioles. OVA + 56 nm nano particles also induced severe airway inflammation, but the severity was less than that of OVA + 14 nm nano particles. Either OVA or nano particles alone resulted in slight recruitment of eosinophils and neutrophils. Vehicle administration caused little infiltration of inflammatory cells.

To quantitate the infiltration of inflammatory cells around the airways, we expressed the number of these cells per length of basement membrane of the airways (Table 2). The number of eosinophils was greater in the OVA group than in the vehicle group without significance. The number of eosinophils was significantly greater in the OVA + 14 nm nano particle group than in the vehicle, the 14 nm nano particle, or the OVA group (P < 0.01). The number was greater also in the OVA + 56 nm nano particle group than in the OVA group or the 56 nm nano particle group, but the difference did not achieve significance. OVA increased the number of neutrophils as compared with vehicle challenge without significance. In the presence of OVA, nano particles with a diameter of 14 nm significantly increased the number as compared with vehicle or OVA challenge (P < 0.01 for vehicle, P < 0.05 for OVA)). In the presence of OVA, nano particles with a diameter of 56 nm increased the number as compared with vehicle (P < 0.05) or OVA (N. S.). Challenge with nano particles increased the numbers as compared with vehicle challenge (P < 0.01 for 14 nm nano particle, N. S. for 56 nm nano particle). There were no significant differences between the OVA + nano particle groups and the nano particle groups. The number of mononuclear cells was significantly greater in the OVA group than in the vehicle group (P < 0.05). 14 nm nano particles significantly increased the number (P < 0.05 versus vehicle). The number was significantly greater in the OVA + 14 nm group than in the OVA group or the 14 nm nano particle group (P < 0.01). The number was also greater in the OVA + 56 nm nano particle group than in the 56 nm nano particle group (P < 0.05) or the OVA group, but the difference did not reach statistical significance.

**Table 2:** Numbers of inflammatory cells and goblet cells in lung tissue.

Group	Animals	Eosinophils	Neutrophils	Mononuclear Cells	Goblet Cells
		
	*(n)*	*(n/mm)*			
vehicle	8	0.187 ± 0.064	0.616 ± 0.140	0.495 ± 0.224	0.177 ± 0.070
14 nm	8	1.419 ± 0.466	3.825 ± 1.073 **	2.431 ± 0.736 *	0.243 ± 0.068
56 nm	7	0.252 ± 0.055	1.710 ± 0.426	0.967 ± 0.418	2.510 ± 2.249
OVA	6	2.442 ± 0.761	1.671 ± 0.222	2.546 ± 0.479 *	5.262 ± 4.150
OVA + 14 nm	6	7.252 ± 2.745 ** ^## $$^	4.144 ± 0.795 ** ^#^	5.393 ± 0.560 ** ^## $$^	17.141 ± 6.702 ** ^# $$^
OVA + 56 nm	6	3.022 ± 0.830	2.546 ± 0.563 *	2.202 ± 1.086	12.932 ± 3.230 * ^$^

Animals received intratracheal instillation of vehicle, nano particles, OVA, or OVA + nano particles for 6 wk. Lungs were removed and fixed 24 h after the last intratracheal administration. Sections were stained with Diff-Quik for measurement of inflammatory cells around the airways or with PAS for goblet cells in the bronchial epithelium. Results are expressed as numbers of cells per length of basement membrane of airways. Values are mean ± SEM. *P < 0.05 versus vehicle, **P < 0.01 versus vehicle, ^#^P < 0.05 versus OVA, ^##^P < 0.01 versus OVA. ^$^P < 0.05 versus nano particles. ^$$^P < 0.01 versus nano particles.

### Nano particles increase goblet cells after antigen challenge

To evaluate airway epithelial injury and hypersecretion of mucus, lung sections were stained with PAS (Table 2). OVA plus 56 nm nano particles increased the number of goblet cells as compared with vehicle without significance. The number was significantly greater in the OVA + 14 nm nano particle group than in the vehicle (P < 0.01), the OVA (P < 0.05), or the 14 nm nano particle group (P < 0.01). The number was greater also in the OVA + 56 nm nano particle group than in the vehicle (P < 0.05), the OVA (N. S.), or the 56 nm nano particle group (P < 0.05).

### Effects of nano particles on local expression of Th2 cytokines in the presence of antigen

To explore the role of local expression of Th2 cytokines in the effects of nano particles on antigen-related airway inflammation, we quantitated protein levels of IL-5, IL-4, and IL-13 in the lung tissue supernatants (Table 3). OVA challenge increased the level of IL-5 as compared with vehicle challenge without significance. In the presence of OVA, nano particles significantly elevated levels of IL-5 as compared with vehicle (P < 0.01) or OVA (P < 0.05 for 56 nm, P < 0.01 for 14 nm). The levels were significantly greater in the OVA + nano particle groups than in the nano particle groups (P < 0.01). The levels of IL-13 were significantly greater in the OVA + 14 nm nano particle group than in the OVA group or 14 nm nano particle group (P < 0.01). The levels were greater also in the OVA + 56 nm nano particle group than in the OVA group (N. S.) or the 56 nm nano particle group (P < 0.05). The level of IL-4 was significantly lower in the OVA + 56 nm nano particle group than in the OVAgroup (P < 0.05). There were no other significant differences among the experimental groups.

**Table 3:** Protein levels of Th2 cytokines in the lung tissue supernatants.

Group	Animals	IL-5	IL-13	IL-4
		
	*(n)*	*(pg/total lung tissue supernatants)*		
vehicle	16	5.5 ± 1.1	4.0 ± 1.1	204.3 ± 13.1
14 nm	13	4.6 ± 1.9	7.4 ± 4.3	194.7 ± 12.8
56 nm	14	7.1 ± 1.9	21.1 ± 8.9	204.3 ± 12.5
OVA	16	26.8 ± 11.1	16.2 ± 7.3	216.5 ± 14.8
OVA + 14 nm	16	113.7 ± 32.0** ^## $^	120.3 ± 42.4** ^## $^	178.5 ± 16.2
OVA + 56 nm	17	88.8 ± 38.0** ^# $^	61.8 ± 27.5*	170.8 ± 17.6^#^

Six groups were intratracheally inoculated with vehicle, nano particles, OVA, or the combination of OVA and nano particles for 6 wk. Lungs were removed and frozen 24 h after the last intratracheal administration. Protein levels in the lung tissue supernatants were analyzed using ELISA. Results are shown as mean ± SEM. *P < 0.05 versus vehicle, **P < 0.01 versus vehicle, ^#^P < 0.05 versus OVA, ^##^P < 0.01 versus OVA. ^$^P < 0.05 versus nano particles. ^$^P < 0.01 versus nano particles.

### Effects of nano particles on local expression of eotaxin, MCP-1, RANTES, and IL-6 in the presence of antigen

To investigate the local expression of eotaxin, MCP-1, RANTES, and IL-6, we measured protein levels of these cytokine and chemokines in the lung tissue supernatants (Table 4). OVA challenge increased the levels of eotaxin without significance as compared with vehicle challenge. The levels were significantly greater in the OVA + nano particle groups than in the vehicle (P < 0.01), the nano particle group (P < 0.05 for OVA + 56 nm nano particle, P < 0.01 for OVA + 14 nm nano particle), or the OVA (P < 0.05 for OVA + 56 nm nano particle, P < 0.01 for OVA + 14 nm nano particle) group. Nano particle challenge increased the levels of MCP-1 as compared to vehicle challenge (P < 0.01 for 14 nm, N. S. for 56 nm). OVA challenge slightly increased the levels without significance as compared with vehicle challenge. Nano particles combined with OVA enhanced the level as compared with nano particle alone (P < 0.01 for 14 nm nano particle, N. S. for 56 nm nano particle) or OVA alone (P < 0.01 for OVA + 14 nm nano particle group, P < 0.05 for OVA + 56 nm nano particle group). The levels of RANTES were significantly greater in the OVA + nano particle groups than in the vehicle group (P < 0.01 for OVA + 14 nm nano particle, P < 0.05 for OVA + 56 nm nano particle), or the OVA group (P < 0.01 for OVA + 14 nm nano particle, P < 0.05 for OVA + 56 nm nano particle), or the nano particle groups (P < 0.01 for 14 nm, N. S. for 56 nm). The levels of IL-6 were significantly greater in the OVA + 14 nm and OVA + 56 nm nano particle groups than in the vehicle group (P < 0.01), the OVA group (P < 0.01 for OVA + 14 nm nano particle group, P < 0.05 for OVA + 56 nm nano particle group), or the nano particle groups (P < 0.01).

**Table 4:** Protein levels of eotaxin, MCP-1, RANTES, and IL-6 in the lung tissuesupernatants.

Group	Animals	eotaxin	MCP-1	RANTES	IL-6
		
	*(n)*	*(pg/total lung tissue supernatants)*			
vehicle	16	67.9 ± 2.9	20.8 ± 3.5	174.6 ± 15.8	105.8 ± 3.6
14 nm	13	99.4 ± 7.1	239.0 ± 17.4**	192.3 ± 18.0	151.7 ± 8.2
56 nm	14	89.5 ± 9.2	89.3 ± 7.4	201.6 ± 22.1	106.5 ± 4.9
OVA	16	130.9 ± 32.3	41.9 ± 9.2	160.7 ± 17.1	118.3 ± 5.7
OVA + 14 nm	16	804.8 ± 175.7** ^## $$^	542.8 ± 45.4** ^## $$^	432.6 ± 27.8** ^## $$^	297.7 ± 30.2** ^## $$^
OVA + 56 nm	17	399.1 ± 86.9** ^# $^	124.0 ± 14.1** ^#^	235.4 ± 16.8* ^#^	202.4 ± 54.2** ^# $$^

Six groups were intratracheally inoculated with vehicle, nano particles, OVA, or the combination of OVA and nano particles for 6 wk. Lungs were removed and frozen 24 h after the last intratracheal administration. Protein levels in the lung tissue supernatants were analyzed using ELISA. Results are shown as mean ± SEM. *P < 0.05 versus vehicle, **P < 0.01 versus vehicle, ^#^P < 0.05 versus OVA, ^##^P < 0.01 versus OVA. ^$^P < 0.05 versus nano particles. ^$$^P < 0.01 versus nano particles.

### Effects of nano particles on 8-OHdG formations in the presence or absence of antigen

**Figure 2:**
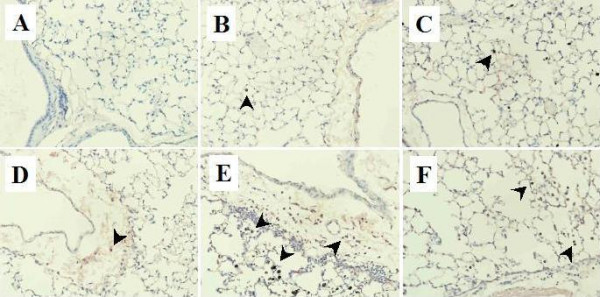
Immunohistological staining for 8-hydroxy-2'-deoxyguanosine (8-OHdG) in the lung obtained from (A) vehicle group, (B) 14 nm nano particle group, (C) 56 nm nano particle group, (D) OVA group, (E) OVA + 14 nm nano particle group, and (F) OVA + 56 nm nano particle group (n = 8 in each group). Lungs were removed twenty-four h after the last intratracheal instillation. Arrows denote positive staining. Original magnification × 300.

We next studied 8-OHdG formation generated from deoxyguanosine in DNA by oxidative stress in the lung. In the vehicle group, nuclear staining with 8-OHdG was barely detectable (Fig. 2A). Nano particles or OVA challenge induced moderate staining with 8-OHdG (Fig. 2B, C, D). On the other hand, OVA plus nano particles resulted in intense immunoreactive 8-OHdG staining as compared to OVA or nano particles alone (Fig. 2E, F). The intensity and the extent of the immunoreactivity were more prominent in the OVA + 14 nm nano particle group (Fig. 1E) than in the OVA + 56 nm nano particle group (Fig. 2F). As typically shown in the OVA + nano particle groups, we found the expression of 8-OHdG in macrophages phagocyting nano particles as well as polymorphonuclear leukocytes (Fig. 2E, F).

We performed morphometric analysis to quantitate the extent and intensity of immunoreactive 8-OHdG among the experimental groups. As compared to vehicle treatment (immunohistochemical score, mean ± SEM: 0.8 ± 0.3), nano particles or OVA treatment revealed increased immunoreactivity (14 nm nano particle: 2.0 ± 0.4, P < 0.05 versus vehicle; 56 nm nano particle: 1.5 ± 0.2; OVA: 1.9 ± 0.5). The scores were greater in OVA + nano particle groups (OVA + 14 nm nano particle group: 2.9 ± 0.6; OVA + 56 nm nano particle group: 2.5 ± 0.3) than in the vehicle (P < 0.01), OVA, or nano particle groups.

### Effects of nano particles on adjuvant activity for total IgE and antigen-specific production of IgG and IgE

To exanime whether nano particles have adjuvant activity for total IgE and antigen-specific Ig production, we measured total IgE and antigen-specific IgG_1_, IgG_2a_, and IgE (Table 5). Total IgE levels were significantly greater in the OVA + nano particle groups than in the vehicle group (P < 0.01 for OVA + 14 nm nano particle group, P < 0.05 for OVA + 56 nm nano particle group). The levels were also significantly greater in the OVA + 14 nm nano particle group than in the OVA or the 14 nm nano particle group (P < 0.05). The antigen-specific IgG_1 _was significantly greater in the OVA + 14 nm nano particle group than in the other groups (P < 0.01 versus each other group). The antigen-specific IgG_2a _was not significantly different among the experimental groups. The combination of OVA plus 14 nm nano particles significantly increased antigen-specific production of IgE as compared with vehicle or OVA alone (P < 0.05).

**Table 5:** Levels of total IgE and antigen-specific IgG1, IgG2a, and IgE.

Group	Animals *(n)*	Total IgE *(ng/ml)*	Antigen-Specific IgG_1 _*(titers)*	Antigen-Specific IgG_2a _*(titers)*	Antigen-Specific IgE *(fluorescene intensity)*
vehicle	12	93.8 ± 27.5	485.3 ± 274.2	152.1 ± 48.8	625.2 ± 49.3
14 nm	9	152.3 ± 36.1	1880.3 ± 1656.2	163.5 ± 49.2	698.7 ± 61.0
56 nm	11	304.1 ± 127.4	1128.2 ± 717.1	182.5 ± 72.9	610.9 ± 54.1
OVA	10	443.4 ± 173.2	2178.5 ± 910.7	256.7 ± 206.2	576.2 ± 89.3
OVA + 14 nm	10	871.9 ± 246.7** ^# $^	28050.3 ± 13840.4** ^## $^	354.9 ± 141.7	860.7 ± 120.5* ^##^
OVA + 56 nm	10	515.6 ± 122.4*	4150.5 ± 1657.0	126.7 ± 32.2	600.7 ± 62.8

Six groups were intratracheally inoculated with vehicle, nano particles, OVA, or the combination of OVA and nano particles for 6 wk. Serum samples were retrieved 24 h after the last intratracheal instillation. Total IgE and antigen-specific IgG_1_, IgG_2a_, and IgE were analyzed using ELISA. Results are expressed as mean ± SEM. *P < 0.05 versus vehicle, **P < 0.01 versus vehicle, ^#^P < 0.05 versus OVA, ^##^P < 0.01 versus OVA, ^$^P < 0.01 versus nano particles.

## Discussion

The present study demonstrated that nano particles administered by the intratracheal route enhanced airway inflammation associated with antigen challenge in mice. The inflammatory component was characterized by increased numbers of eosinophils, neutrophils, and mononuclear cells. Recruitment of these cells was accompanied by an increment in goblet cells in the bronchial epithelium. The airway inflammation induced by the combined administration of nano particles with antigen modulated local expression of IL-5, eotaxin, IL-13, RANTES, MCP-1, and IL-6. The formation of 8-OHdG was moderately induced by nano particles or antigen alone, and was further enhanced by antigen plus nano particles as compared with nano particles or antigen alone. The enhancing effects were more prominent with 14 nm nano particles than with 56 nm nano particles. Furthermore, 14 nm nano particles enhanced total IgE and antigen-specific production of IgG_1 _and IgE.

DEP exacerbate allergic diseases including allergic asthma [5]. Elementary carbon, which is mainly involved in the nuclei of DEP, can enhance allergic sensitization. We used CB in the present study, since CB is a useful prototypical particle for the research on the effects and their mechanisms of PM including DEP. Because CB is relatively inert, the effects of particle size can be elucidated without confounding factors [23]. Al-Humadi and coworkers have demonstrated that CB exacerbates airway inflammation related to antigen in rats [18]. Last and colleagues have demonstrated that ambient particles with a diameter of less than 2.5 μm partially exacerbated lung inflammation related to antigen [17]. However, the comparative study focusing on the effects of particle size on antigen-related airway inflammation has never been conducted *in vivo*. In the present study, nano particles aggravated antigen-related airway inflammation, which was confirmed by the counts of inflammatory leukocytes in BAL fluid and by the histological assesment. Furthermore, we showed that nano particles exaggerated goblet cell hyperplasia elicited by antigen. In overall trends, the enhancing effects were more prominent with 14 nm nano particles than with 56 nm nano particles. Furthermore, 14 nm nano particles had obvious adjuvant activity for the antigen-specific production of IgG_1 _and IgE. These results clearly indicate that nano particles can aggravate antigen-related airway inflammation *in vivo*. Also, the effects are greater with smaller particles than with larger particles. We have previously examined the effects of DEP on allergic airway inflammation using 100 μg of DEP *in vivo *[5,24,25]. Based on the previous studies from our laboratory, we chose the dosage of 50 μg/body of nano particles, which can be considered to be involved in 100 μg of DEP as elementary carbon. Indeed, the enhancing effects of 14 nm nano particles on the airway inflammation and cytokine expressions are comparable to those of DEP in the previous study [5]. Another important point in this study is the surface area of the nano particles used. Surface area of particles exposed reportedly correlates magunitude of airway inflammation [26]. In our study, the surface area of the 14 nm nano particles was 6.7 fold larger than that of 56 nm nano particles (300 m^2^/g versus 45 m^2^/g). Nano particles with larger surface area are likely to attach more immunoregulative molecules than those with smaller surface area. As a result, smaller nano particles (14 nm) may lead to more prominent aggravation of antigen-related airway inflammation than larger nano particles (56 nm) in the present study. We did not examine the effects of the nano particles with the same particle number in the present study. However, the number of smaller nano particles is larger than that of larger nano particles when the particles make the same weight. Alternatively, our study has demonstrated not only the size effects of nano particles, but also the effects of their surface area and/or the effects of their number on the antigen-related airway inflammation. Future independent studies uniforming the surface area or particle number will provide better widestanding for the effects of the nano particles on antigen-related airway inflammation.

Allergic asthma is often associated with activation of IL-5 gene cluster, a pattern compatible with predominant activation of Th2-like T-lymphocyte population. IL-5 is essential for maturation of eosinophils in the bone marrow and their release into the blood [27,28]. Also, these Th2 cytokines are implicated in the pathogenesis of allergic reactions via their roles in mediating IgG_1 _and IgE production, and in differentiation, vascular adhesion, recruitment, activation, and survival of eosinophils. In our study, airway inflammation induced by the combined administration of nano particles and antigen were concomitant with the increased protein levels of IL-5. These results provide the first evidence that nano particles can accelerate antigen-related IL-5 expression and subsequent eosinophilic inflammation.

IL-13 is also recognized to regulate eosinophilic inflammation, and mucus secretion [29]. On the other hand, IL-6 is believed to participate in airway remodeling [30]. In the present study, nano particles enhanced the expression of the proteins in the presence of antigen. Therefore, nano particles may aggravate mucus hypersecretion and airway remodeling, at least partly, through the enhanced expression of IL-13 and IL-6. In fact, the OVA + nano particle groups showed enhancement in the mucus hypersecretion as compared with the OVA group or the nano particle groups.

Among chemokines, eotaxin is essential for eosinophil recruitment in antigen-related airway inflammation [31,32]. RANTES is a strong chemotactic and activating factor for eosinophils, and can modulate eosinophil adhesion [33,34]. In fact, our previous studies have confirmed that the exaggerated allergic airway inflammation induced by DEP paralleled the local elevation of the inflammatory proteins [5,22]. In the present study, nano particles enhanced the expression of these proteins in the presence of antigen as compared with antigen alone. The results suggest that nano particles aggravate allergic airway inflammation, at least in part, via the enhancement of the local expression of these proteins.

Interestingly, in our study, nano particles challenge increased the lung level of MCP-1 as compared to vehicle challenge. Also, the levels were significantly greater in the OVA + nano particle groups than in the vehicle or the OVA group. MCP-1 is a CC chemokine, and is chemoattractant for monocytes [34]. It also has a chemoattractant effect of CD4^+ ^and CD8^+ ^T lymphocytes [35]. MCP-1 also plays a role in recruitment of eosinophils to acute and chronic inflammatory sites [36]. Furthermore, some particles such as silica [37] and amosite asbestos [38] reportedly can induce MCP-1 *in vitro *and *in vivo*. Thus, our findings indicate that pulmonary exposure to carbon nano particles may induce MCP-1 expression in the airways. In addition, the aggravating effects of nano particles on antigen-related airway inflammation should be mediated, at least in part, via the enhanced expression of this chemokine.

In overall trends, the enhancing effects of nano particles on local expression of cytokines and chemokines related to antigen challenge were more prominent with 14 nm nano particles than with 56 nm nano particles. The differences in the enhanced expression of the proteins between the two sizes of nano particles may contribute, at least partly, to the differences in the magnitude of antigen-related airway inflammation and goblet cell hyperplasia.

Redox imbalance is a critical factor for tissue injury in various pulmonary diseases including inflammation such as asthma [39,40]. Airway macrophages from individuals with asthma produce more reactive oxidative species (ROS) than those from control subjects [41]. On the other hand, nano particles have been implicated to induce and/or enhance oxidative stress [42]. Furthermore, a recent study has demonstrated that the same type of nano particles as we used can induce ROS in the alveolar macrophages [43]. We, therefore, evaluated the contribution of oxidative stress to the deterious effects of nano particles on antigen-related airway inflammation. 8-OHdG is a proper marker of the oxidative stress. In our study, immunoreactivity of 8-OHdG in the lung was more intense in the OVA + nano particle groups than in the nano particle groups or the OVA group. Further, enhanced immunoreactivity for 8-OHdG was detected in alveolar macrophages as well as polymorphonuclear leukocytes. It is suggested that the enhanced oxidative stress is involved, at least partly, in the aggravation of antigen-related airway inflammation caused by nano particles.

Antigen-specific IgE is thought to contribute to inflammatory cell accumulation after antigen challenge via degranulation of mast cells [44]. On the other hand, IgG with antigen is a strong agonist for eosinophil degradation *in vitro *[45]. Furthermore, late asthmatic reactions are associated with IgG antibody [46]. Previous studies have reported that DEP with antigen demonstrate adjuvant activity for IgE production *in vivo *[47] and that those without antigen induce nonspecific IgE response in humans [48,49]. In addition, we have reported that DEP enhance antigen-specific production of IgE and IgG induced by intratracheal challenge with antigen *in vivo *[5,25]. In the present study, the combined intratracheal administration of 14 nm nano particles and antigen showed a significant greater increase in total IgE and antigen-specific IgG_1 _and IgE than the other administration. The enhancement in the antigen-specific immunogloblin production by 14 nm nano particles can induce the enhanced release of a variety of inflammatory mediators such as histamine and leukotrienes, resulting in aggravated manifestations of allergic asthma.

Finally, in the real world, we inhale nano particles and antigen in ambient air, not particle suspension nor aliquot of antigen. The dose of nano particles injected in the present study can be estimated to be less than a hundred fold than that we inhale in daily life. Further, real PM including DEP are complex mixture of carbon, metals, and organics, which are different from CB used in the present study. Thus, it remains to be elucidated in future whether daily inhalation of nano particles with or without other compounds including organic chemicals than elementary carbon combined with occasional exposure of aerosol antigen lead to the same results as the present study.

## Conclusion

The present study has shown evidence that nano particles can aggravate antigen-related airway inflammation. The effect may be mediated, at least partly, through the increased local expression of IL-5 and eotaxin and also by the modulated expression of IL-13, MCP-1, IL-6, and RANTES. Furthermore, 14 nm nano particles enhance total IgE and antigen-specific production of IgG_1 _and IgE. These results suggest that nano particles can be a risk for exacerbation of allergic asthma. The aggravating effect may be larger with the smaller particles.

## Competing interests

The author(s) declare that they have no competing interests.

## Authors' contributions

KI participated in the design of the study and collection of the data, performed the statistical analysis and wrote initial drafts of the manuscript. HT participated in the design of the study, helped to organize the data and the results, and to prepare the manuscript. RY, MS, and KS participated in the collection of the data. TI participated in the design and coordination and helped to draft the manuscript. TY helped to prepare the manuscript. All authors read and approved the final manuscript.
